# Supplementary material for: Polyphenols from Maackia amurensis Heartwood Protect Neuronal Cells from Oxidative Stress and Prevent Herpetic Infection
Source: Int J Mol Sci. 2024 Apr 9;25(8):4142. doi: 10.3390/ijms25084142 (PMC11050087; doi:10.3390/ijms25084142)
Supplement: Supplementary file 1 [file ijms-25-04142-s001.zip › ijms-2941411-supplementary.pdf]

Supplementary data

Polyphenols from *Maackia amurensis* heartwood protect neuronal cells from oxidative stress and prevent herpetic infection

Darya V. Tarbeeva[1,*], Evgeny A. Pislyagin[1], Ekaterina S. Menchinskaya[1], Dmitrii V. Berdyshev[1], Natalya V. Krylova[2], Olga V. Iunikhina[2], Anatoliy I. Kalinovskiy[1], Mikhail Y. Shchelkanov[2], Natalia P. Mishchenko[1], Dmitry L. Aminin[1], and Sergey A. Fedoreyev[1]

[1] G.B. Elyakov Pacific Institute of Bioorganic Chemistry, Far-Eastern Branch of the Russian Academy of Science, 690022 Vladivostok, Russia; tarbeeva1988@mail.ru (D.V.T.); pislyagin_ea@piboc.dvo.ru (E.A.P.); menchinskaya_es@piboc.dvo.ru (E.S.M.); berdyshev@piboc.dvo.ru (D.V.B.); kaaniw@piboc.dvo.ru (A.I.K.); daminin@piboc.dvo.ru (D.L.A.); fedoreev-s@mail.ru (S.A.F.)

[2] G.P. Somov Institute of Epidemiology and Microbiology, Rospotrebnadzor, 690087, Vladivostok, Russia; krylovanatalya@gmail.com (N.V.K.); olga_iun@inbox.ru (O.V.I.); adorob@mail.ru (M.Y.S.)

*Correspondence: tarbeeva1988@mail.ru (D.V.T.); Tel.: +79143446441

File S1. Quantum-chemical modeling

All quantum-chemical calculations were done using B3LYP and cam-B3LYP exchange-correlation functionals, the polarization continuum model (PCM) and 6-311G(d) basis set implemented in Gaussian 16 package of programs[1].

The statistical weights (g_{im}) of individual conformations were calculated according to equation 1:

$$g_{im} = e^{-\Delta G_{im}/RT} \Big/ \sum_i e^{-\Delta G_{im}/RT} \qquad (1)$$

where $\Delta G_{im} = G_i - G_m$ are the relative Gibbs free energies and index "*m*" denotes the most stable conformation.

The ECD spectra were calculated using time-dependent density functional theory (TDDFT), cam-B3LYP functional, PCM model and 6-311G(d) basis set for conformations, which relative Gibbs free

[1] Frisch, M.J.; Trucks, G.W.; Schlegel, H.B.; Scuseria, G.E.; Robb, M.A.; Cheeseman, J.R.; Scalmani, G.; Barone, V.; Petersson, G.A.; Nakatsuji, H.; Li, X.; Caricato, M.; Marenich, A.V.; Bloino, J.; Janesko, B.G.; Gomperts, R.; Mennucci, B.; Hratchian, H.P.; Ortiz, J.V.; Izmaylov, A.F.; Sonnenberg, J.L.; Williams-Young, D.; Ding, F.; Lipparini, F.; Egidi, F.; Goings, J.; Peng, B.; Petrone, A.; Henderson, T.; Ranasinghe, D.; Zakrzewski, V.G.; Gao, J.; Rega, N.; Zheng, G.; Liang, W.; Hada, M.; Ehara, M.; Toyota, K.; Fukuda, R.; Hasegawa, J.; Ishida, M.; Nakajima, T.; Honda, Y.; Kitao, O.; Nakai, H.; Vreven, T.; Throssell, K.; Montgomery, J.A.; Jr.; Peralta, J.E.; Ogliaro, F.; Bearpark, M.J.; Heyd, J.J.; Brothers, E.N.; Kudin, K.N.; Staroverov, V.N.; Keith, T.A.; Kobayashi, R.; Normand, J.; Raghavachari, K.; Rendell, A.P. ; Burant, J.C.; Iyengar, S.S.; Tomasi, J.; Cossi, M.; Millam, J.M.; Klene, M.; Adamo, C.; Cammi, R.; Ochterski, J. W.; Martin, R.L.; Morokuma, K.; Farkas, O.; Foresman, J.B.; Fox, D.J. Gaussian 16, Revision A.03; Gaussian, Inc.: Wallingford. CT, USA, **2016**.

energies satisfy to relation $\Delta G_{im} \leq 4$ kcal/mol. The seventy-five electronic transitions were calculated for each conformation of scirpusin A (**13**) and ninety-five – for maackiazin (**10**). The bandwidths $\zeta = 0.30$ eV and the UV shifts $\Delta\lambda = +17$ nm were used for Gauss-type functions, taken for simulating the individual bands in theoretical spectra.

The scaled theoretical and experimental ECD spectra were obtained according to equation:

$$\Delta\varepsilon_{sc}(\lambda) = \Delta\varepsilon(\lambda) \Big/ \left|\Delta\varepsilon(\lambda_{peak})\right| \qquad (2)$$

where the denominator $|\Delta\varepsilon(\lambda_{peak})|$ is a modulo of the peak value for the most intensive negative characteristic band in region200 $\leq \lambda \leq 240$ nm in corresponding ECD spectrum.

NMR spin-spin coupling constants were calculated using GIAO approach and "mixed" keyword.

The theoretical statistically averaged ECD spectra containe low-intensive bands at $\lambda \geq 250$ nm. This fact pointes out, that averaging must be done on the basis of a more complicated theoretical model, then the model used in our study. For example, one-dimensional cyclic potential for the internal rotation of the anisole substituent in **10** containes four minima. In the present study the ECD spectra were calculated only for these four equilibrium configurations and their statistical weights were calculated using rigid-rotor-harmonic-oscillator approximation. The more rigorous model must account for nonequilibrium configurations and averaging of the ECD spectra must be done using vibrational distribution function calculated for this LAM process. Analogous improvements may be performed for other LAM processes.

Table S1. ^{1}H (700 MHz) NMR data for compound **10** (δ in ppm, J in Hz, acetone-d_6).

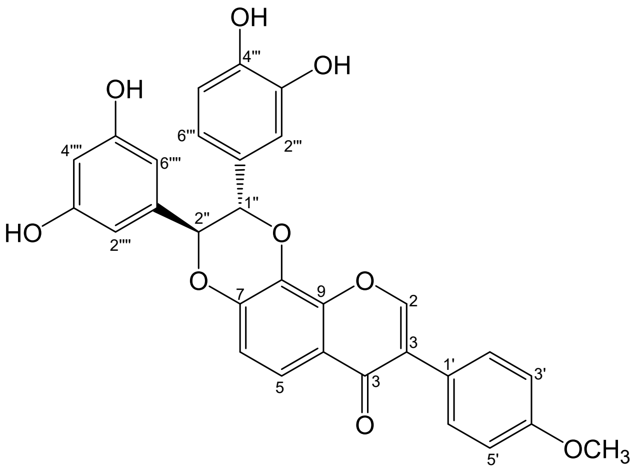

Position	^{1}H
2	8.23, s, 1H
5	7.74, d, J = 8.6, 1H
6	7.09, d, J = 8.6, 1H
1', 6'	7.58, dd, J = 8.7, 2.2, 2H
2', 5'	7.00, dd, J = 8.7, 2.2, 2H
1''	5.07, d, J = 7.9, 1H
2''	5.03, d, J = 7.9, 1H
2'''	6.84, d, J = 2.0, 1H
5'''	6.75, d, J = 8.1, 1H
6'''	6.65, dd, J = 8.1, 2.0, 1H
2'''', 6''''	6.33, d, J = 2.1, 2H
5''''	6.31, d, J = 2.1, 1H
OCH_3-4'	3.84, s, 3H

Table S2. ^{1}H (700 MHz) NMR data for compound **13** (δ in ppm, J in Hz, acetone-d_6).

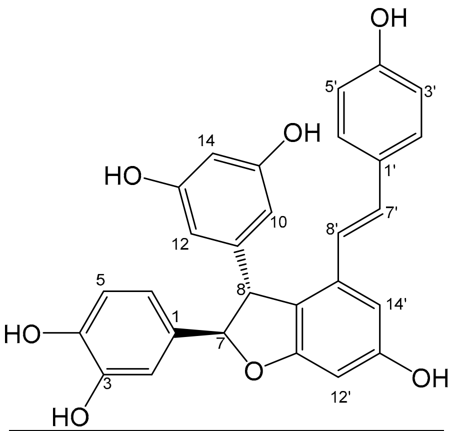

Position	^{1}H
1	6.70, dd, J = 8.1, 2.0, 1H
2	6.80, d, J = 8.1, 1H
6	6.82, d, J = 2.0, 1H
7	5.35, d, J = 5.3, 1H
8	4.44, d, J = 5.3, 1H
10, 12, 14	6.23, s, 3H
2', 6'	7.17, dd, J = 8.6, 2.0, 1H
3', 5'	6.73, dd, J = 8.6, 2.0, 1H
7'	6.90, d, J = 16.1, 1H
8'	6.72, d, J = 16.1, 1H
12'	6.71, d, J = 2.0, 1H
14'	6.32, d, J = 2.0, 1H

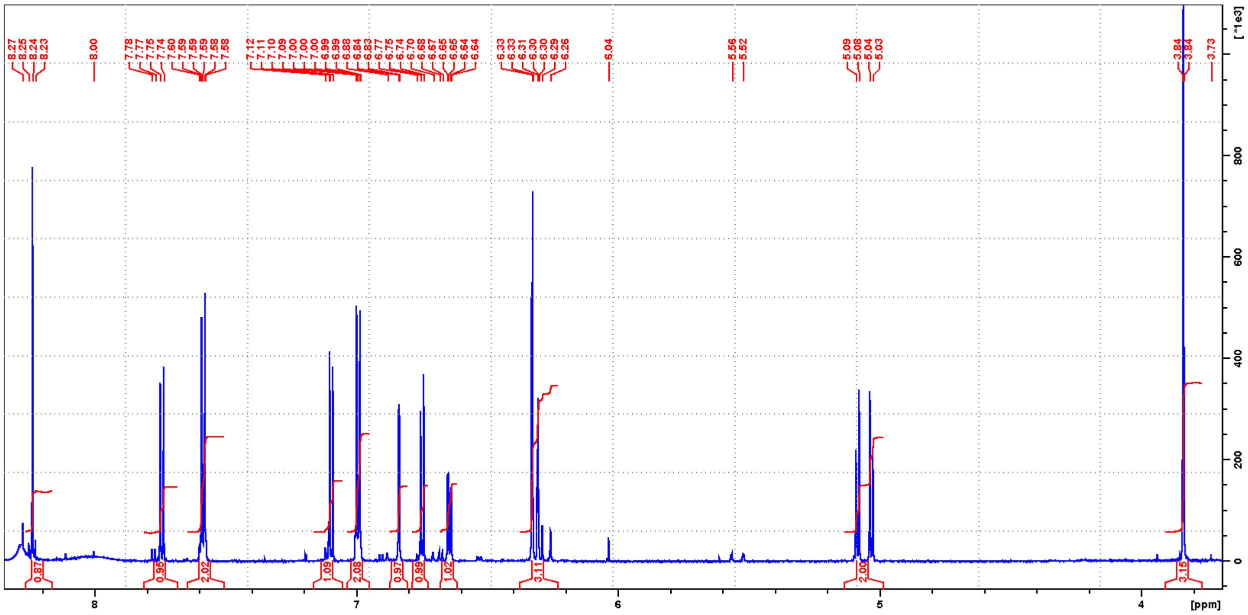

Figure S1. ^{1}H (700 MHz) NMR spectrum for compound **10** (δ in ppm, J in Hz, acetone-d_6).

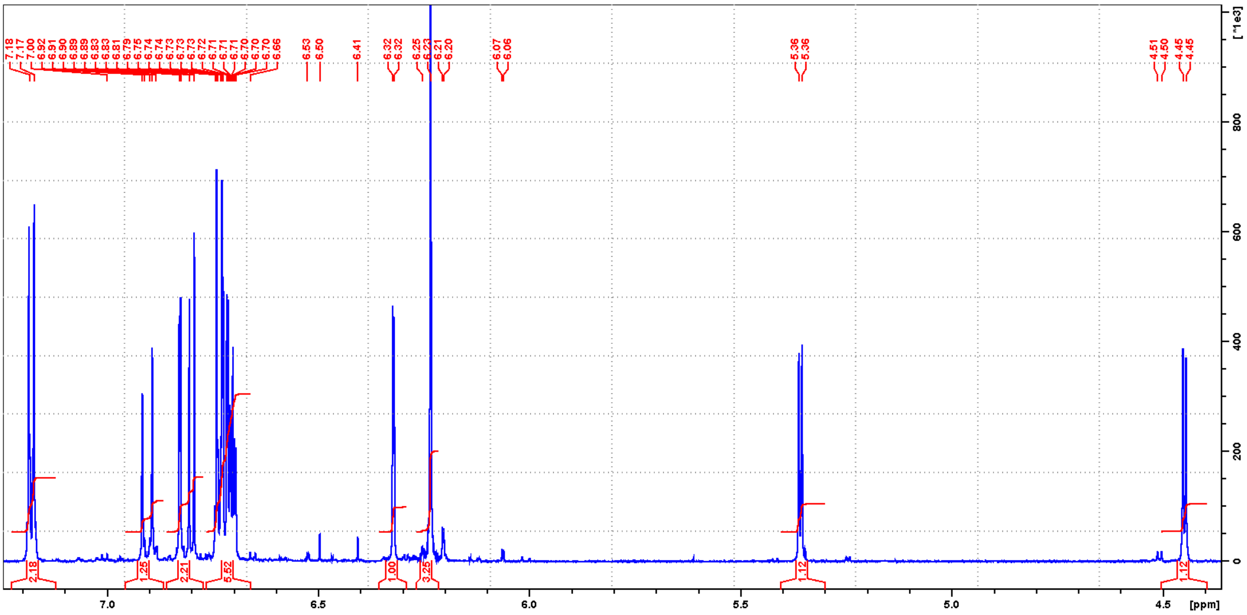

Figure S2. ^{1}H (700 MHz) NMR spectrum for compound **13** (δ in ppm, J in Hz, acetone-d_6).

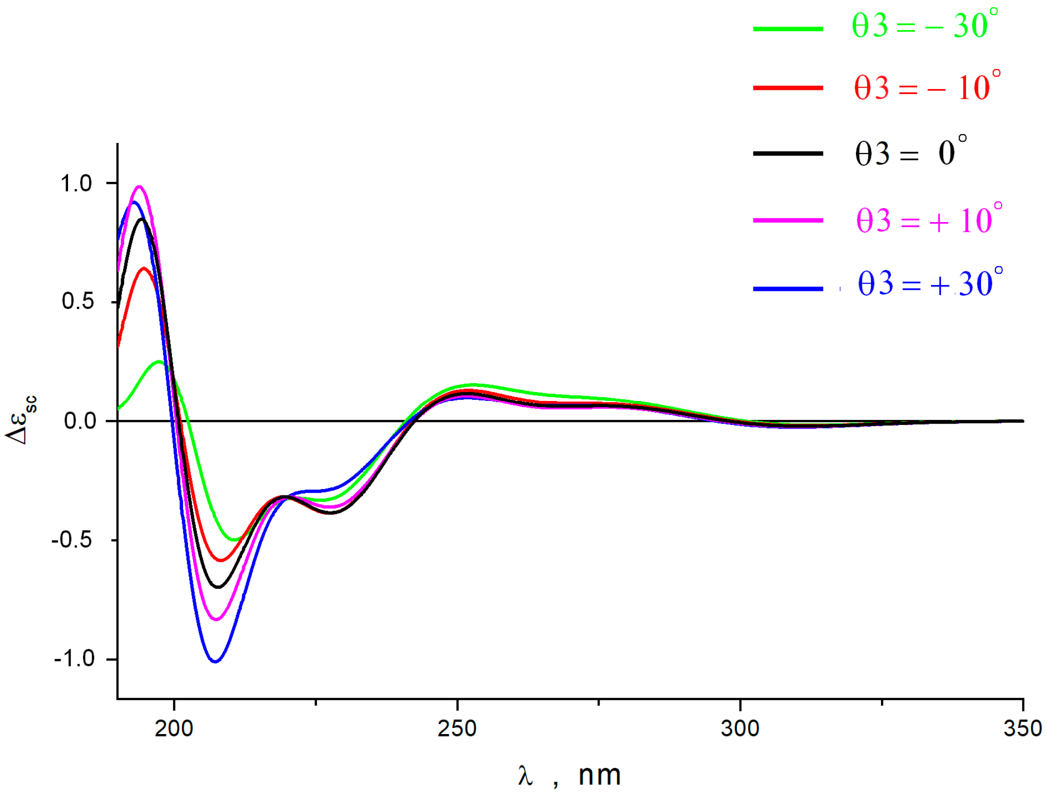

Figure S3. ECD spectra, calculated for different values of dihedral angle θ3 ≡ ∠C2'''–C1'''–C1''–H1''.

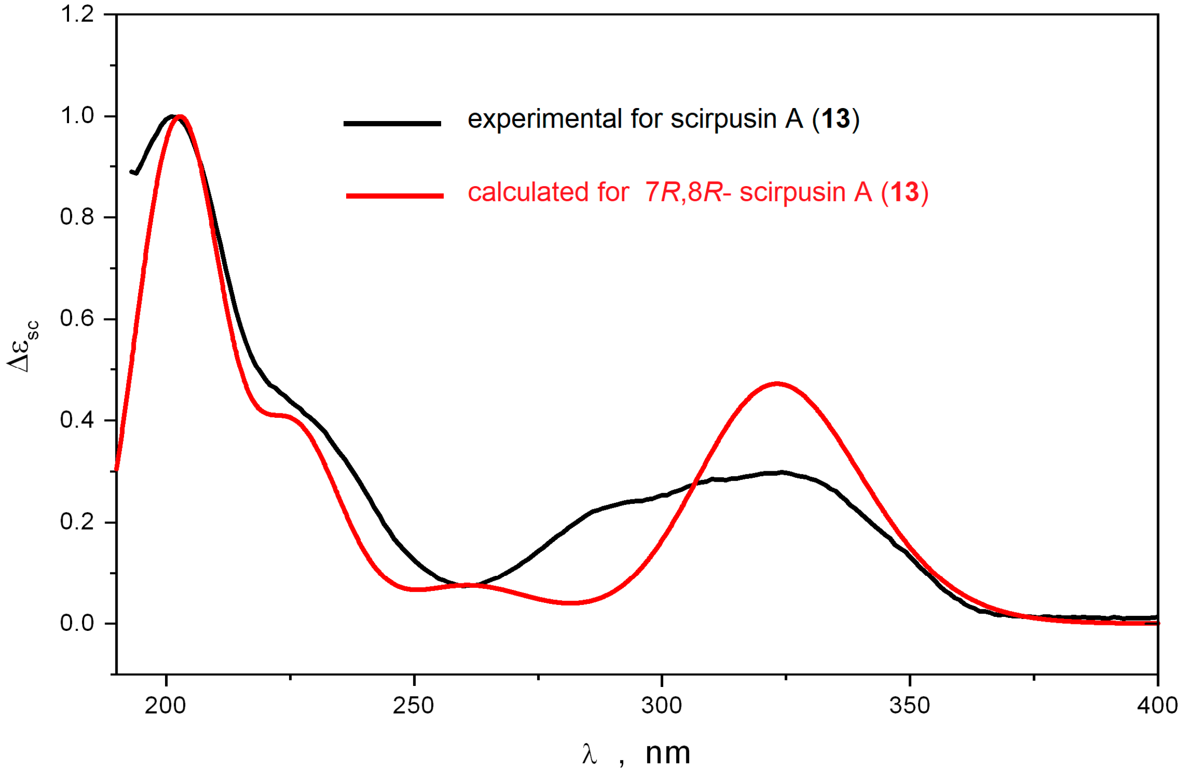

Figure S4. Calculated and experimental UV spectra for scirpusin A (**13**).

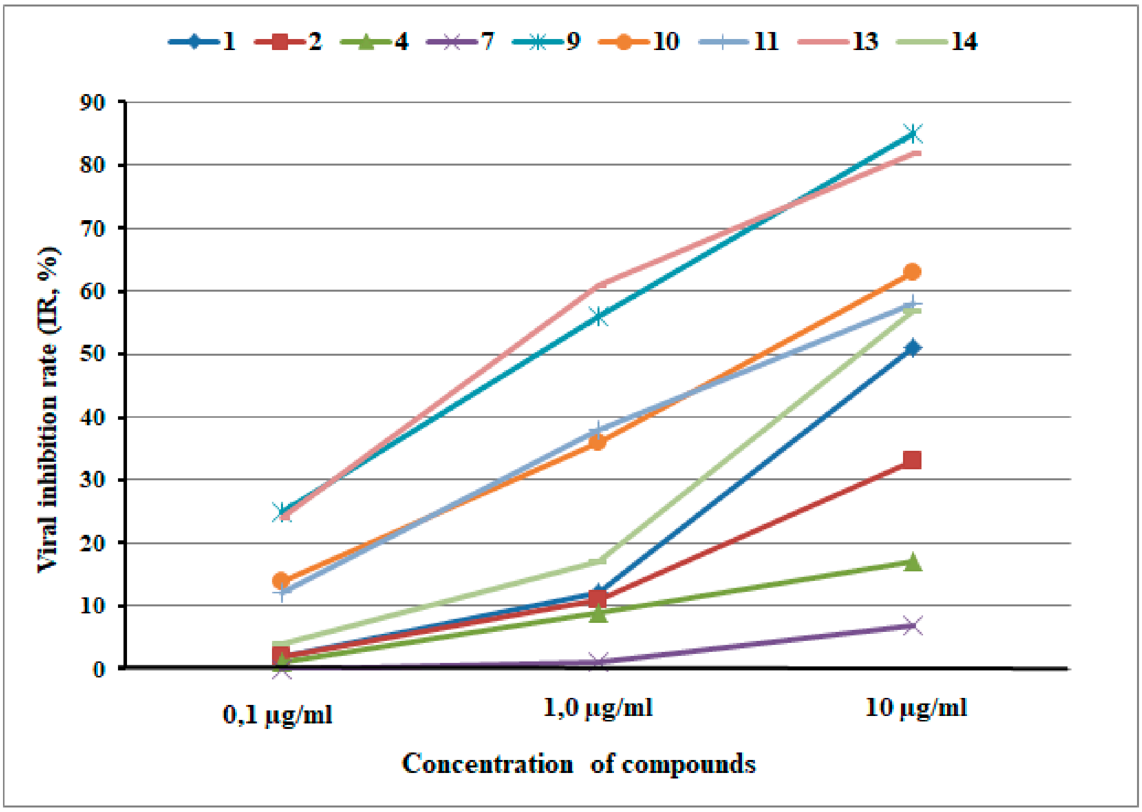

Figure S5. Inhibitory effect ofpolyphenolsduringpretreatment of HSV-1 with compounds.

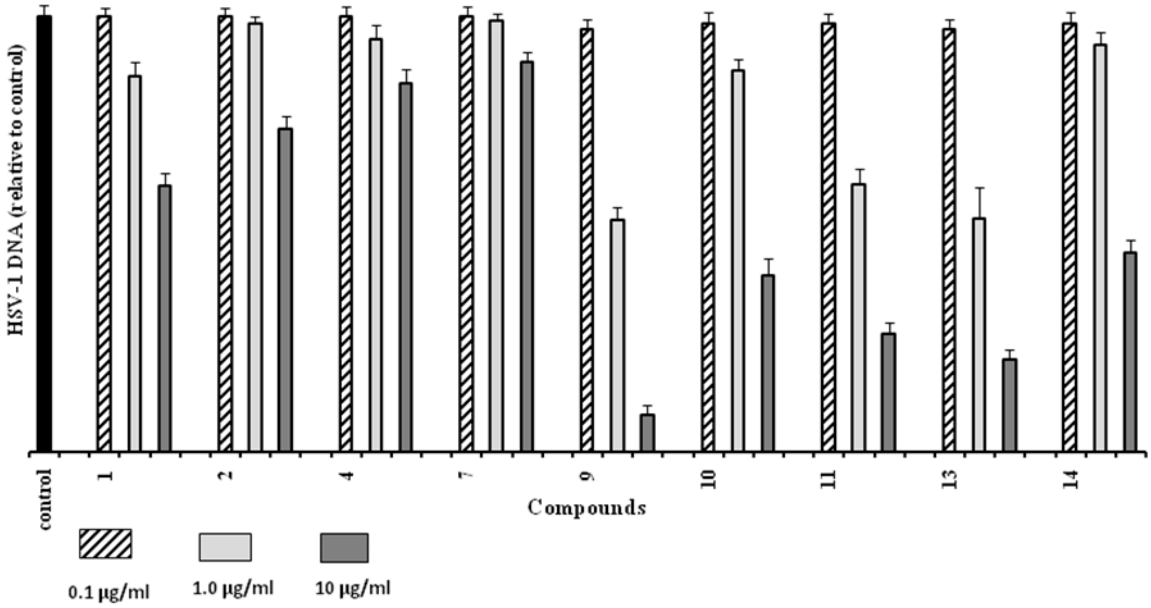

Figure S6. Virucidal activity of polyphenolic compounds (RT-PCR assay, dose-dependant graphs)
